# Book Review

**Published:** 1948

**Authors:** 


### Reviews of Books

**Treatment of Some Chronic and Incurable Diseases.** By A. T. TODD, O.B.E., M.B., M.R.C.P. Second Edition. Pp. xii, 324. Bristol: John Wright & Sons Ltd. 1947. 25s.—This is a second edition of a book first published in 1937. There is an unmistakable French influence, borne out by the references. French medicine has much to its credit, but its heyday was in the last century and the highly clinical approach which is so characteristic has often been to blame for the continued application of the term "incurable" to so many disorders. Most of the arguments are too facile, based on too slender a foundation of fact, and frequently incomprehensible. There is much careless phraseology : e.g. the lady "who was a daily vegetable laxative addict of high dosage," or the pain which "may be felt along the lower left dorsal vertebrae." Duodenitis and "Hepatic Defect" are obviously regarded as playing an important, if not almost central, role, the former being found in "thirty per cent. of investigated sick people." Anyone who wants to know how to fill the day and empty the bowel of a constipated duchess should find the book useful.

**Diseases of the Chest.** By MAURICE DAVIDSON, M.D., F.R.C.P. Third Edition. Pp. xvi, 670. Illustrated. London : Oxford University Press (Geoffrey Cumberlege). 1948. 50s.—This already well-established book is approaching its middle-age spread, with an increase of nearly 100 pages in the latest edition. The arrangement of the text is pleasing, the illustrations with plates and radiographs are liberal and admirably reproduced, and there is a useful selected bibliography. The size of the book and the careful viewing of subjects from different angles, with the avoidance of too much dogma, makes it more suitable for the post-graduate than the undergraduate student. Nevertheless, it is for the latter a most commendable and authoritative reference volume. The book is a practical manual based on the author's wide experience and illustrated by many case reports. Thanks to the most readable style in which it is presented, it is easy to appreciate the wisdom and sound principles of medicine as well as the detailed information which it contains.

**Changing Disciplines.** By JOHN A. RYLE, M.D. Pp. xvi, 124. London : Oxford University Press. 1948. 12s. 6d.—This book contains the subject-matter of lectures given on the history, method and motives of social pathology. The author gives due praise to the public-health reformers of the past and shows how a development of their ideas and principles has led to a newer and more humanistic approach to medicine. This is a balanced interpretation which illustrates the value and the importance of social medicine for the medical student. It is the best book on the subject which we have yet seen.

**Tuberculosis in Childhood.** By DOROTHY S. PRICE. M.D., Second Edition. Pp. vii, 219. Illustrated. Bristol : John Wright & Sons Ltd. 1948. 25s.—A clear description is given of all the clinical types of tuberculous disease in infancy and adolescence. Stress is rightly placed upon prevention and early diagnosis. The student will find it easy to follow the argument that tuberculosis is potentially a generalizing disease in early life, and every opportunity should be given for the healing of primary lesions. The discussion on B.C.G. can be regarded as a very useful guide to its possible uses in this country. The clinical descriptions of miliary tuberculosis and tuberculous meningitis are specially welcome in view of the present possibilities of effective streptomycin treatment. This book is a compendium of good clinical material, sense and judgment, with emphasis on the art of diagnosis. It is satisfactorily illustrated with radiographs and photographs : there is an up-to-date bibliography and clear references.

**The Mechanism of Abdominal Pain.** By V. J. KINSELLA, M.B.Ch.M., F.R.C.S., F.R.A.C.S. Pp. xi, 230. Illustrated. Sydney: Angus & Robertson Ltd. 1948. Price 32s. 6d.—This book is the outcome of an honest attempt to give a basis for abdominal pain, relying on human experiment only; the fundamental inadequacy of animal-pain experiments is emphasized. The author's theory is that the effective stimulus for pain is a raised tension within the tissues, and that the afferent fibres for this pass up via the sympathetic nervous system. A number of human clinical experiments are described, and the literature on the subject has obviously been carefully studied and analysed. We have come to expect sound reasoning, unbiased by previous theories, from this author, and we are certainly not disappointed in this book. Whether the passage of time, and further investigation, will prove his theories correct or not, remains to be seen. In any case, it is a most stimulating and thought-provoking book.

**Radiotherapy and Cancer.** By A. G. C. TAYLOR, M.R.C.S., L.R.C.P., D.R.; JOAN LASSETTER, M.B., CH.B., D.R., and T. K. MORGAN, M.B., M.R.C.S., D.M.R. (T.). Pp. 81. London: H. K. Lewis & Co. Ltd. 1948. Price 7s. 6d.—The fact that this little book is being circulated to the general practitioners working within the area of the Wessex Radiological Board will make a big step towards the much needed co-operative understanding between radiotherapist and his most important ally. Part I will afford them a useful, brief survey of the principles of radiotherapy practised by Taylor and his colleagues. Part II, with the exception of the frank " not seen by us ", reminds one of " Aids . . . ", and should the participants in the largest part of the circulation succeed in their primary diagnosis, they may then refer for a very brief survey of pathology, policy and prognosis. Nevertheless, the book ably contributes towards filling the gap for those whose limited time prevents their reading one of the larger works on Radiotherapy.

